# A 10-year comparative analysis of medical and surgical specialty lobbying by physician professional organizations

**DOI:** 10.1093/haschl/qxaf140

**Published:** 2025-07-09

**Authors:** Max Bouvette, Stephanie Beveridge, Kirtana Kumar, Mehak Ali, Justin Dvorak, Nirmal Choradia, Ryan Nipp

**Affiliations:** The University of Oklahoma College of Medicine, The University of Oklahoma Health Sciences Center, Oklahoma City, OK 73104, United States; The University of Oklahoma College of Medicine, The University of Oklahoma Health Sciences Center, Oklahoma City, OK 73104, United States; The University of Oklahoma College of Medicine, The University of Oklahoma Health Sciences Center, Oklahoma City, OK 73104, United States; The University of Oklahoma College of Medicine, The University of Oklahoma Health Sciences Center, Oklahoma City, OK 73104, United States; The University of Oklahoma Health Sciences Center, Department of Biostatistics and Epidemiology, Oklahoma City, OK 73104, United States; Section of Hematology/Oncology, Department of Medicine, Stephenson Cancer Center and The University of Oklahoma Health Sciences Center, Oklahoma City, OK 73104, United States; Section of Hematology/Oncology, Department of Medicine, Stephenson Cancer Center and The University of Oklahoma Health Sciences Center, Oklahoma City, OK 73104, United States

**Keywords:** lobbying, health policy, physicians

## Abstract

**Introduction:**

Physician professional organizations (PPOs) engage in lobbying to advocate for their interests and influence health policy. However, trends in lobbying across specialties are not well characterized. Disproportionate spending across PPOs may affect the ability to shape healthcare legislation and ensure that all physician voices are represented.

**Methods:**

We analyzed publicly available lobbying data from OpenSecrets.org covering 2014-2023, adjusted to 2023 USD. A total of 109 PPOs were included. Physician professional organizations were categorized as medical (*n* = 68), surgical (*n* = 29), or overlapping (*n* = 12), based on whether they primarily represented medical specialties, surgical specialties, or both. Physician workforce data from the AAMC were used to calculate spending per-physician. Temporal trends were assessed using a Mann-Kendall test.

**Results:**

Median annual PPO lobbying spending was $71 million, with a significant downward trend (*P* < .01, tau = −.64). Expenditures included $32 million (45%) by medical PPOs, $12 million (16%) by surgical PPOs, and $27 million (39%) by overlapping PPOs. The median annual lobbying spending per-physician was $78.

**Conclusion:**

These findings suggest that PPOs have not sustained lobbying investments over time, particularly when accounting for the effects of inflation.

## Introduction

Healthcare lobbying seeks to influence legislation that affects patients, physicians, and medical practice.^1-4^ Health professional groups, including physician professional organizations (PPOs), often engage in lobbying to shape policymaker decisions and advocate for their respective fields' interests.^5^ Among PPOs, the American Medical Association (AMA), which represents the broader physician community, leads in lobbying spending, with lobbying investments of ∼$21 million in 2023 alone.^6,7^ In contrast, many specialty-specific organizations focus more narrowly on issues pertinent to their respective field(s). Political engagement by PPOs is important, especially given the increasing complexity of medical care and the financial toxicity associated with treatment of certain medical conditions.^8-10^ These challenges are compounded by persistent barriers to care, including the complexities of the US health insurance system.^11-16^ While physician advocacy can play a valuable role in advancing healthcare delivery, the interests of physicians and the broader public may not always perfectly align.

A limited number of studies have evaluated political activities within the health sector, with some focus on expenditures of the pharmaceutical industry and healthcare professionals.^17-19^ Previous literature highlights a significant overall increase in health sector lobbying expenditures since 2000, with the pharmaceutical industry leading spending at $100-$400 million annually, an amount consistently 2-4 times larger than the annual spending by other contributing industries such as hospital/nursing homes, health services/HMOs, or health professionals.^5,8,19^ However, little information has been reported on lobbying activities by PPOs, with limited evidence suggesting that their spending has remained stable or potentially declined.^5,19^ Recent work has described an increase in lobbying expenditures among oncology-related groups, indicating potential for variation among different medical fields and specialties.^5,20^ To date, a dearth of research exists assessing the overall spending patterns of PPOs, including consideration of variance across different specialty types. Physician professional organizations rely on lobbyists who possess expertise in navigating the political climate and communicating with government officials.^21^

In the current study, we sought to identify and describe trends in PPOs' lobbying spending over the last 10 years (from 2014-2023). Specifically, we aimed to describe healthcare lobbying spending and lobbying spending per-physician over time, with particular interest in comparing medical vs surgical specialty organizations. We hypothesized that surgical specialty PPOs would demonstrate higher lobbying on a per-physician basis, as these specialties are generally higher earning.^22^ By providing a detailed description of PPOs' lobbying spending and political activities, we hope to inform broader efforts to quantify the impact of physician advocacy and bring attention to recent shifts in lobbying expenditures.

## Methods

### Data sources

This study was deemed exempt from Institutional Board Review (IRB) review due to the use of publicly available, de-identified data that did not meet criteria of human subject research. We extracted publicly available lobbying data provided annually by OpenSecrets (https://www.opensecrets.org), with a focus on expenditures by PPOs from the years 2014-2023. Organizations are not required to disclose the specific allocation of their lobbying funds, which likely range across lobbyist salaries, staff expenses, payments to firms, consulting, research, and other expenses. We did not extract any data related to political action committees or campaign contributions, which were outside the scope of our study. The Association of American Medical Colleges (AAMC) provides the total number of physicians biennially within the AAMC Physician Specialty Data Report and AAMC U.S Physician Workforce Data Dashboard.^23,24^ We utilized the available physician datasets to calculate lobbying spending on a per-physician basis across the years 2014-2023. The AAMC does not provide data on specialties with less than 2500 active physicians and only reports data biennially, therefore we performed linear extrapolation on available data, deriving a line of best fit (values available for 5/10 total years, alternating from 2014-2023). The resulting models were used to predict the number of physicians for missing years, allowing for continuous annual estimates.

### Inclusion criteria and PPO categorization

Inclusion criteria for PPOs were as follows: (1) the group was physician-led and primarily composed of physicians; (2) the group was nationally representative; and (3) the group was not a commercial entity, private practice, or coalition composed of multiple organizations. A total of 109 PPOs spent lobbying dollars over the period (2014-2023). Multiple investigators independently reviewed each PPO and categorized them into specialty and practice type (medical, surgical, or overlapping). We resolved discrepancies through discussion among the team and consensus with a third investigator. Physician professional organizations representing more than 1 unique specialty were designated as “multiple.” Among the 109 PPOs that reported lobbying expenditures from 2014 to 2023, 68 were classified as medical, 29 as surgical, and 12 as overlapping. The overlapping category included PPOs not exclusively belonging to a medical or surgical specialty but rather representing physicians in both areas of practice (eg, AMA and American Academy of Pediatrics). Table S1 documents each PPO's classification by specialty and practice type (medical vs surgical vs overlapping) to ensure replicability of future work.

We referenced physician workforce datasets available from the AAMC to create a comprehensive list of specialties with at least 2500 active physicians. In the categorization process, the following were treated as medical specialties: Allergy and Immunology, Anesthesiology, Cardiology, Dermatology, Emergency Medicine, Endocrinology, Family Medicine, Gastroenterology, Geriatric Medicine, Hematology and Oncology, Infectious Diseases, Internal Medicine, Internal Medicine/Pediatrics, Nephrology, Neurology, Pain Management, Pathology, Pediatrics, Physical Medicine and Rehabilitation, Preventative Medicine, Psychiatry, Pulmonary Disease/Critical Care, Radiation Oncology, Radiology/IR, and Rheumatology. In the categorization process, the following were treated as surgical specialties: General Surgery, Neurological Surgery, Obstetrics and Gynecology, Ophthalmology, Orthopedic Surgery, Otolaryngology, Plastic Surgery, Thoracic Surgery, Urology, and Vascular Surgery.

To ensure a completeness, we reviewed additional sectors within the health industry and identified 7 PPOs that, although not exclusively listed under the Health Professionals category, clearly met our inclusion criteria. These organizations were subsequently added to the sample: Infectious Diseases Society of America, Society of Hospital Medicine, American Academy of Sleep Medicine, American Academy of Home Care Medicine, Community Oncology Alliance, American Society of Metabolic and Bariatric Surgery, and the American College of Sports Medicine.

### Variables and measures

The specific variables we extracted from OpenSecrets.com under the health sector and health professional industry included lobbying spending across all health professional groups and lobbying spending by all PPOs (data collected as of April 2024). We determined annual lobbying expenditures across 2014-2023 for each category of interest (all health professionals, all PPOs, medical specialty PPOs, surgical specialty PPOs, and overlapping specialty PPOs). We adjusted dollar values to January 2023 US dollars using the Consumer Price Index inflation calculator.^25^ Lobbying spending on a per-physician basis was also calculated to account for differences that could be attributed to specialty size. To determine spending per-physician, we divided the lobbying spending from each category of PPOs by their respective size as provided by the AAMC.

### Statistical analysis

To describe temporal trends in lobbying, we used a Mann-Kendall test, with a significance level set to .05. The test yielded a *P*-value for significance and a tau value ranging from −1 to 1 describing trend direction. We performed statistical analysis using SPSS software version 29. All datasets used for analysis were freely available via download from the OpenSecrets website.

## Results

**Figure 1.**
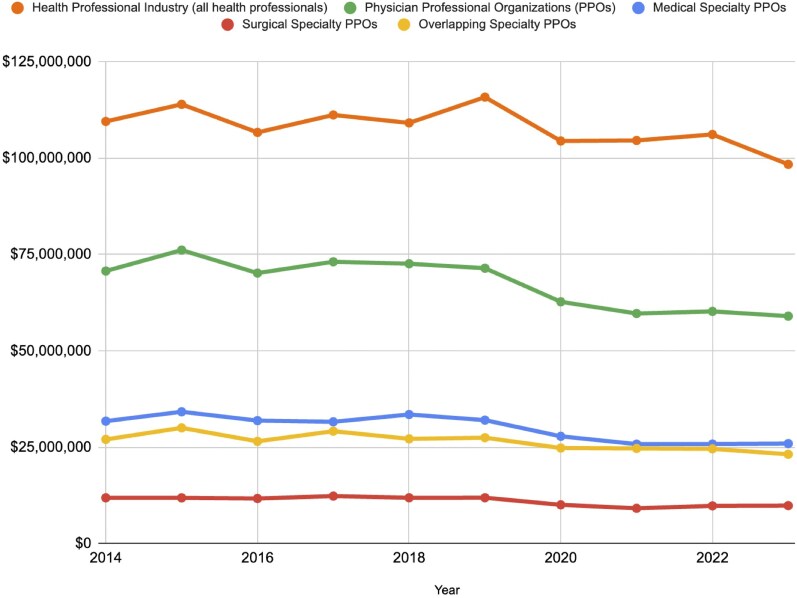
Temporal trends in lobbying spending by health professional groups—including breakdown of physician professional organizations.

From 2014-2023, overall health sector lobbying spending increased from $627.2 million up to $745.4 million, with a peak in 2021 at $808.5 million. Annual lobbying spending within the health sector demonstrated a significant positive trend in spending over time (tau = .78, *P* < .01) (Table 1). Health sector spending consisted of lobbying listed under several areas, including pharmaceuticals/health products, hospitals/nursing homes, health services/HMOs, and the health professional industry. We then determined temporal trends in lobbying spending across the health professional industry, including all PPOs, medical specialty PPOs, surgical specialty PPOs, and overlapping specialty PPOs (Table 1, Figure 1).

**Table 1.** Lobbying spending over time by the health sector, health professional industry, and PPOs by category.

Year	Overall health sector lobbying spending	Health professional industry lobbying spending (includes PPOs)	Overall (both medical and surgical) PPOs lobbying spending	Medical specialty PPOs lobbying spending	Surgical specialty PPOs lobbying spending	Overlapping specialty PPOs lobbying spending
2014	$627.2	$109.5	$70.7	$31.8	$11.9	$27.0
2015	$656.2	$114.0	$76.2	$34.2	$11.9	$30.0
2016	$653.0	$106.7	$70.2	$31.9	$11.7	$26.5
2017	$696.8	$111.2	$73.1	$31.6	$12.3	$29.2
2018	$693.1	$109.1	$72.6	$33.5	$11.9	$27.2
2019	$728.4	$115.8	$71.5	$32.0	$11.9	$27.5
2020	$743.9	$104.5	$62.7	$27.9	$10.1	$24.8
2021	$808.5	$104.6	$59.7	$25.8	$9.2	$24.7
2022	$786.2	$106.1	$60.2	$25.8	$9.8	$24.6
2023	$745.4	$98.4	$59.0	$26.0	$9.9	$23.2
Median	$712.6	$107.9	$70.5	$31.7	$11.8	$26.8
*P*-value	*P* < .01	*P* = .06	*P* < .01	*P* = .04	*P* = .09	*P* < .01
Z (trend)	+0.78	−0.47	−0.64	−0.51	−0.42	−0.64

Dollars are inflation adjusted to 2023 values and reported in millions USD.

Lobbying expenditures by health professional groups totaled $107.9 million per year (10-year median). The industry showed a negative trend in lobbying spending over the period, but this change did not meet statistical significance (tau = −.47, *P* = .06). Sixty-five percent (65%) of the total health professional group lobbying spending was accounted for by PPOs ($70.5 million/$107.9 million). The remaining 35% came from non-physician health professional groups, commercial groups, private practices, and coalitions which did not meet our PPO inclusion criteria (Table 1). Further, overall lobbying spending by PPOs had a significant downward trend over time (tau = −.64, *P* < .01). Within PPOs, lobbying spending by medical specialty organizations totaled $31.7 million per year (45%) (10-year median). Moreover, lobbying spending by surgical specialty organizations totaled $11.8 million per year (16%) (10-year median). Physician professional organizations in the overlapping category contributed the remaining $26.8 million per year (39%) (10-year median). Medical specialty organizations showed a significant downward trend over time in spending (tau = −.51, *P* = .04). Lobbying by surgical specialty organizations did not significantly change, although they showed a similar downward trend (tau = −.42, *P* = .09).

**Figure 2.**
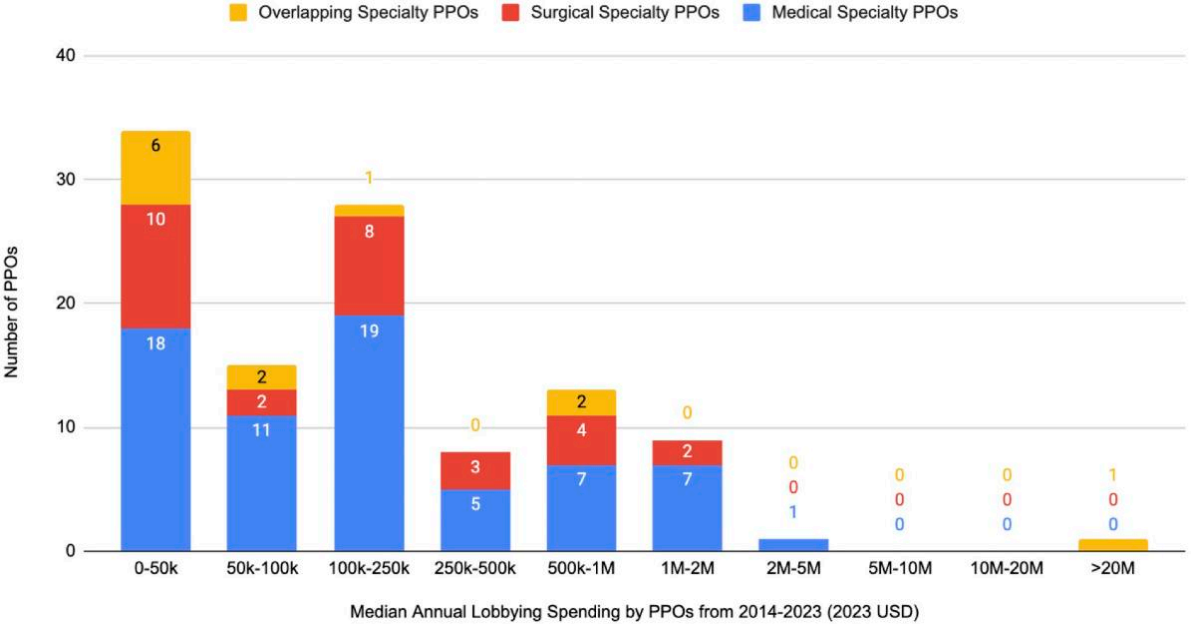
Histogram showing distribution of median annual lobbying spending by physician professional organizations (2014-2023).

Importantly, lobbying spending was not evenly distributed, as a small number of organizations contributed the majority of spending. For instance, we found the AMA represented ∼30%-35% of PPOs annual spending, an amount 5-7 times greater than the next highest spender within the health professional industry. Of note, the AMA was included in our analysis as an overlapping specialty PPO given their broad representation of physicians in the United States. In Figure 2, we present a histogram of anonymized annual spending across PPOs, treating all years without recorded expenditures as $0 to account for inactivity or organizational changes. The majority of PPOs (77/109; 70.6%) had a median annual spending of less than $250 000. The distribution shown within our histogram highlights the skewed nature of physician lobbying, with a small subset of organizations responsible for a large amount of spending.

From 2014-2023, the total number of physicians in the United States increased from 843 791 to 998 591 (+18.4%, *P* < .01), with an average annual growth of 1.9% (Table S2). On average, 73% of physicians (671 266) practiced in medical specialties and 16% (146 099) in surgical specialties. The remaining 12% were not included in AAMC specialty-level reporting due to small specialty size or unclassified roles. Although both groups grew significantly over time (*P* < .01), medical specialties expanded more (+15.5%) than surgical specialties (+5.9%).

We used these physician workforce data to determine lobbying spending on a per-physician basis for our study period. Physician professional organizations' lobbying spending on a per-physician basis was $78 per year for all physicians, $48 per year for medical specialists, and $81 per year for surgeons (10-year medians) (Table 2). Lobbying spending per physician significantly decreased for all 3 groups across the period (*P* < .01). We could not provide per-physician spending for the overlapping category, due to an inability to reliably determine the number of physicians represented.

**Table 2.** Lobbying spending by PPOs on a per-physician basis.

Year	PPOs lobbying spending per-physician—overall	PPOs lobbying spending per-physician—medical specialties	PPOs lobbying spending per-physician—surgical specialties
2014	$83.82	$51.16	$83.53
2015	$88.45	$54.02	$82.64
2016	$79.93	$49.68	$81.16
2017	$81.90	$48.51	$85.29
2018	$79.57	$50.46	$81.46
2019	$76.10	$47.23	$80.48
2020	$66.25	$40.64	$68.05
2021	$62.87	$37.83	$62.90
2022	$60.90	$36.07	$64.43
2023	$59.11	$36.19	$65.41
Median	$77.83	$47.87	$80.82
*P*-value	*P* < .01	*P* < .01	*P* < .01
Z (trend)	−0.91	−0.82	−0.69

Calculations performed using data from the AAMC Physician Specialty Data Report. Overlapping specialty PPOs are not reported due to inability to determine the associated number of physicians. Dollars are inflation adjusted to 2023 values and reported in millions USD.

## Discussion

In this study, we sought to provide the first comprehensive report on the lobbying activities of PPOs. Our results underscore an overall rising health sector lobbying expenditure, a significant trend that is interestingly not reflected by health professional groups and PPOs. Total spending by the health professional industry did not demonstrate a significant change, and lobbying spending by PPOs exhibited a significant downward trend over time. While medical specialty PPOs spent more overall, surgical specialty PPOs had a higher spending on a per-physician basis. Our results highlight that PPO spending is unevenly distributed and has lagged behind inflation in recent years. Collectively, our work contributes novel data describing the state of healthcare lobbying efforts.

Although prior studies have broadly analyzed political activities of the health sector and pharmaceutical industry, less attention has focused on health professional groups.^17,18^ The limited work reporting on lobbying activities of PPOs suggests that spending has either remained stable or potentially decreased.^5,19^ Lobbying spending is disproportionally concentrated among a small number of PPOs, carrying important implications for the physician community. The strategic consolidation of resources under larger organizations may enhance advocacy efforts, but potentially leaves smaller specialties underrepresented. A more complete understanding of these lobbying changes may be instrumental in (1) identifying unique factors that influence lobbying by PPOs, (2) evaluating physicians' ability to influence health policy, and (3) illustrating how these trends affect the healthcare landscape including patient care, affordability, and equitable distribution of resources. Physician organizations often advocate for issues related to professional autonomy, reimbursement models, and clinical practice standards. While these efforts are often rooted in clinical expertise and professional judgment, they may not always align with broader system-level goals such as cost containment or workforce flexibility. For example, PPOs may resist scope-of-practice expansions for advanced practice providers or oppose certain compensation reforms. This acknowledgment is important for understanding the broader impact of physician influence on health policy.

This study's implications include evidence supporting an existing difference in overall and per-physician lobbying spending between medical vs surgical specialties. Across the study period, we observed growth in the physician workforce, with surgical specialties expanding at a slower rate than that of the medical specialties. Despite clear growth in the physician workforce, we found a decline in PPOs overall lobbying spending, thereby bringing to light new potential discussions regarding influence on policy. Discussions surrounding the entire physician workforce become increasingly relevant as the shortage has yet to be wholly addressed.^26-29^

According to the Bureau of Health Workforce, a part of the Health Resources and Services Administration, a shortage of 139 940 full-time equivalent physicians is projected for 2035, noting significant variation across specialties.^26^ With this, lobbying efforts to expand medical programs and residency positions may become increasingly important. Physicians face a variety of evolving pressures, such as increasing regulations, complex payment models, and corporate consolidation.^30-33^ Compared to other stakeholders in the health sector, PPOs represent a smaller share of total lobbying especially when contrasted with hospitals/nursing homes, health services/HMOs, and particularly pharmaceutical companies. This discrepancy suggests a wide variation in financial resources and influence within the health sector. Our work supports the need to better understand recent shifts in lobbying spending to ensure that physicians and PPOs maintain an influence on health policy.

Our findings provide valuable new insights regarding the landscape of health lobbying and physician advocacy; however, several limitations of this study warrant discussion. Lobbying spending reports mandatorily filed with the Clerk of the U.S. House of Representatives and Secretary of the U.S. Senate often lack granularity.^34^ These reports contain general issue codes and items written by submitters, but they frequently offer little to no information on how funds are distributed across the listed issues.^35^ Consequently, while lobbying activities are documented, tracking and/or discerning the specific allocation of funds is difficult. As a result of this limitation, our attempts to elucidate the reasoning and mechanisms behind the observed spending changes became very challenging. Improved transparency in lobbying activities may enhance PPOs' accountability to members and stakeholders. The exclusion of commercial groups, private practices, and coalitions likely limits our ability to completely understand shifts in lobbying expenditures. Our study also includes data from years within the COVID-19 pandemic, during which unique barriers to lobbying efforts arose, potentially influencing our findings.^36-38^ Lobbying firms have no requirement to report information on clients that have spent less than $3000 in a quarter.^39^ Firms with in-house lobbyists can also provide self-estimates rounded to the nearest $10 000.^39^ Based upon these aspects of reporting, the lobbying listed on OpenSecrets may underestimate the actual spending. Finally, we recognize that all lobbying dollars do not provide the same impact, as advocacy outcomes often rely on a complex interplay of public opinion and the political climate.

## Conclusion

This study describes trends in lobbying by PPOs over the past decade. While total lobbying across the health sector increased, spending by physician organizations declined in both total and per physician terms. Medical specialties accounted for the largest share of total PPO lobbying, while surgical specialties had higher spending when adjusted for workforce size. These patterns reflect variation in advocacy across specialties. Improved transparency in lobbying disclosures may help clarify how priorities are determined and may also facilitate investigative efforts to better understand physicians' influence on health policy.

## Supplementary Material

qxaf140_Supplementary_Data
