# Supplementary material for: A 10-year comparative analysis of medical and surgical specialty lobbying by physician professional organizations
Source: Health Aff Sch. 2025 Jul 9;3(7):qxaf140. doi: 10.1093/haschl/qxaf140 (PMC12287694; doi:10.1093/haschl/qxaf140)
Supplement: qxaf140_Supplementary_Data [file qxaf140_supplementary_data.zip › Supplemental Material.docx]

**Supplemental Table 1. Classification of Physician Professional Organizations (PPOs) by Specialty Domain and Practice Type.** Organizations were categorized as medical, surgical, or overlapping based on their primary or dominant specialty focus. PPOs representing more than one specialty were marked as “Multiple.” Organizations with multiple specialties but a single category (e.g., all medical) were still classified as medical or surgical accordingly.

| **Physician Professional Organization (PPO)** | **Primary Specialty (or Multiple)** | **Category (Medical, Surgical, Overlapping)** |
| --- | --- | --- |
| Academy of Allergy & Asthma in Primary Care | Allergy and Immunology | Medical |
| Amer Soc Metabolic & Bariatric Surgery | Multiple | Surgical |
| American Academy of Addiction Psychiatry | Psychiatry | Medical |
| American Academy of Allergy Asthma & Immunology | Allergy and Immunology | Medical |
| American Academy of Child & Adolescent Psychiatry | Psychiatry | Medical |
| American Academy of Dermatology Assn | Dermatology | Medical |
| American Academy of Emergency Medicine | Emergency Medicine | Medical |
| American Academy of Family Physicians | Family Medicine | Medical |
| American Academy of Home Care Medicine | Multiple | Medical |
| American Academy of Neurology | Neurology | Medical |
| American Academy of Ophthalmology | Opthalmology | Surgical |
| American Academy of Orthopaedic Surgeons | Orthopedic Surgery | Surgical |
| American Academy of Otolaryngology | Otolaryngology | Surgical |
| American Academy of Pediatrics | Pediatrics | Overlapping |
| American Academy of Physical Medicine | Physical Medicine and Rehabilitation | Medical |
| American Academy of Sleep Medicine | Multiple | Medical |
| American Assn for Geriatric Psychiatry | Psychiatry | Medical |
| American Assn for Thoracic Surgery | Thoracic Surgery | Surgical |
| American Assn Neuromuscular/Electrodiagnostic Med | Neurology | Medical |
| American Assn of Neurological Surgeons | Neurological Surgery | Surgical |
| American Assn of Physician Specialists | Multiple | Overlapping |
| American Assn/Clinical Endocrinologists | Endocrinology, Diabetes, and Metabolism | Medical |
| American Board of Internal Medicine | Internal Medicine | Medical |
| American Clinical Neurophysiology Society | Neurology | Medical |
| American Coll of Occupational/Enviro Med | Preventative Medicine | Medical |
| American College of Cardiology | Cardiology | Medical |
| American College of Emergency Physicians | Emergency Medicine | Medical |
| American College of Foot & Ankle Surgeon | Orthopedic Surgery | Surgical |
| American College of Gastroenterology | Gastroenterology | Medical |
| American College of Lifestyle Medicine | Multiple | Medical |
| American College of Obstetricians & Gynecologists | Obstetrics and Gynecology | Surgical |
| American College of Osteopathic Family Physicians | Family Medicine | Medical |
| American College of Osteopathic Internists | Internal Medicine | Medical |
| American College of Physicians | Internal Medicine | Medical |
| American College of Preventive Medicine | Preventative Medicine | Medical |
| American College of Radiation Oncology | Radiation Oncology | Medical |
| American College of Radiology | Radiology/IR | Medical |
| American College of Rheumatology | Rheumatology | Medical |
| American College of Sports Medicine | Multiple | Medical |
| American College of Surgeons | General Surgery | Surgical |
| American Gastroenterological Assn | Gastroenterology | Medical |
| American Geriatrics Society | Geriatric Medicine | Medical |
| American Glaucoma Society | Opthalmology | Surgical |
| American Medical Assn | Multiple | Overlapping |
| American Medical Women's Assn | Multiple | Overlapping |
| American Neurological Assn | Neurology | Medical |
| American Osteopathic Assn | Multiple | Overlapping |
| American Psychiatric Assn | Psychiatry | Medical |
| American Soc Cataract/Refractive Surgery | Opthalmology | Surgical |
| American Soc/Tropical Medicine/Hygiene | Infectious Diseases | Medical |
| American Society for Blood & Marrow Transplant | Hematology and Oncology | Medical |
| American Society for Clinical Pathology | Pathology | Medical |
| American Society for Dermatologic Surgery | Dermatology | Surgical |
| American Society for Gastro Endoscopy | Gastroenterology | Medical |
| American Society for Mohs Surgery | Dermatology | Surgical |
| American Society for Radiation Oncology | Radiation Oncology | Medical |
| American Society for Reproductive Medicine | Multiple | Surgical |
| American Society for Transplant/Cellular Therapy | Multiple | Medical |
| American Society of Addiction Medicine | Psychiatry | Medical |
| American Society of Anesthesiologists | Anesthesiology | Medical |
| American Society of Bariatric Physicians | General Surgery | Overlapping |
| American Society of Breast Surgeons | General Surgery | Surgical |
| American Society of Clinical Oncology | Hematology and Oncology | Medical |
| American Society of Echocardiography | Multiple | Medical |
| American Society of Hematology | Hematology and Oncology | Medical |
| American Society of Interventional Pain Physicians | Multiple | Medical |
| American Society of Nephrology | Nephrology | Medical |
| American Society of Nuclear Cardiology | Multiple | Medical |
| American Society of Pediatric Nephrology | Nephrology | Medical |
| American Society of Plastic Surgeons | Plastic Surgery | Surgical |
| American Society of Retina Specialists | Opthalmology | Surgical |
| American Society of Transplant Surgeons | General Surgery | Surgical |
| American Society of Transplantation | Multiple | Overlapping |
| American Thoracic Society | Pulmonary Disease and Critical Care | Medical |
| American Urogynecologic Society | Obstetrics and Gynecology | Surgical |
| American Urological Assn | Urology | Surgical |
| Arthroscopy Assn of North America | Orthopedic Surgery | Surgical |
| Association of Academic Physiatrists | Physical Medicine and Rehabilitation | Medical |
| Association of Black Cardiologists | Cardiology | Medical |
| Association of Hip & Knee Surgeons | Orthopedic Surgery | Surgical |
| Child Neurology Society | Neurology | Medical |
| College of American Pathologists | Pathology | Medical |
| Community Oncology Alliance | Hematology and Oncology | Medical |
| Congress of Neurological Surgeons | Neurological Surgery | Surgical |
| Endocrine Society | Endocrinology, Diabetes, and Metabolism | Medical |
| Infectious Diseases Society of America | Infectious Diseases | Medical |
| Joint Council of Allergy & Immunology | Allergy and Immunology | Medical |
| Kidney Care Council | Nephrology | Medical |
| National Assn of EMS Physicians | Emergency Medicine | Medical |
| National Hispanic Medical Assn | Multiple | Overlapping |
| North American Soc/Pediatric Gastroent | Gastroenterology | Medical |
| North American Spine Society | Multiple | Surgical |
| Obesity Medicine Assn | Multiple | Overlapping |
| Outpatient Ophthalmic Surgery Society | Opthalmology | Surgical |
| Physicians Advocacy Institute | Multiple | Overlapping |
| Physicians for American Healthcare Access | Multiple | Overlapping |
| Renal Physicians Assn | Nephrology | Medical |
| Society for Cardiovasculr Angiography/Intervention | Cardiology | Medical |
| Society for Maternal-Fetal Medicine | Obstetrics and Gynecology | Surgical |
| Society for Neuro-Oncology | Multiple | Medical |
| Society for Vascular Surgery | Vascular Surgery | Surgical |
| Society of Cardiovascular Computed Tomography | Multiple | Medical |
| Society of General Internal Medicine | Internal Medicine | Medical |
| Society of Gynecologic Oncology | Obstetrics and Gynecology | Overlapping |
| Society of Hospital Medicine | Internal Medicine | Medical |
| Society of Interventional Radiology | Radiology/IR | Medical |
| Society of Nuclear Medicine | Radiology/IR | Medical |
| Society of Thoracic Surgeons | Thoracic Surgery | Surgical |
| US Women's Health Alliance | Obstetrics and Gynecology | Surgical |

**Supplemental Table 2.** Total Number of Physicians in Medical and Surgical Specialties per AAMC Physician Specialty Data Report and U.S. Physician Workforce Data Dashboard

| Year | Overall # Physicians | # Physicians in Medical Specialties | # Physicians in Surgical Specialties | Other, specialties with <2500 physicians (not provided by AAMC) |
| --- | --- | --- | --- | --- |
| 2014 | 843,791 | 621,265 | 142,531 | 79,994 |
| 2015 | 860,939 | 633,401 | 143,901 | 83,637 |
| 2016 | 878,191 | 642,638 | 144,387 | 91,165 |
| 2017 | 892,856 | 651,679 | 144,735 | 96,442 |
| 2018 | 912,591 | 664,011 | 146,244 | 102,336 |
| 2019 | 938,980 | 678,520 | 148,130 | 112,330 |
| 2020 | 946,991 | 685,384 | 148,100 | 113,507 |
| 2021 | 949,658 | 682,608 | 145,955 | 121,095 |
| 2022 | 989,320 | 716,559 | 152,206 | 120,555 |
| 2023 | 998,591 | 717,443 | 150,884 | 130,264 |
| Median | 925,786 | 671,266 | 146,099 | 107,333 |
| Percent Increase (2014-2023) | +18.4% | +15.5% | +5.9% | +62.8% |
| p-value | p<0.01 | p<0.01 | p<0.01 | p<0.01 |
